# Association of Elevated Lipoprotein(a) Levels with Major Adverse Cardiovascular Events in Non-Diabetic Patients with Acute Myocardial Infarction: A Cohort Study from Bosnia and Herzegovina

**DOI:** 10.3390/medsci14020227

**Published:** 2026-04-30

**Authors:** Mesud Jamaković, Armin Šljivo, Azra Durak-Nalbantić, Farid Ljuca, Mugdim Bajrić, Behija Hukeljić-Berberović

**Affiliations:** 1 Clinic for Diseases of the Heart, Vessels and Rheumatism, Clinical Center of University Sarajevo, Bolnička 25, 71000 Sarajevo, Bosnia and Herzegovina; dr_jamakovic@yahoo.com (M.J.); azradurak@yahoo.com (A.D.-N.); 2 Clinic for Cardiovascular Surgery, Clinical Center of University Sarajevo, Bolnička 25, 71000 Sarajevo, Bosnia and Herzegovina; behija.berberovic@ssst.edu.ba; 3 Department of Physiology, Faculty of Medicine, University of Tuzla, Univerzitetska 1, 75000 Tuzla, Bosnia and Herzegovina; farid.ljuca@untz.ba; 4 Clinic for Invasive Cardiology, University Clinical Center Tuzla, 75000 Tuzla, Bosnia and Herzegovina; dr.mugdim.bajric@gmail.com

**Keywords:** lipoprotein(a), acute myocardial infarction, coronary artery disease, major adverse cardiovascular events, SYNTAX score

## Abstract

Background/Objectives: Elevated lipoprotein(a) [Lp(a)] is an independent causal risk factor for atherosclerotic cardiovascular disease and may contribute to increased coronary complexity and adverse outcomes after acute myocardial infarction (AMI). Data regarding its prognostic significance in Southeastern Europe remains limited. This study aimed to evaluate the association between elevated Lp(a) levels, coronary artery disease severity, and major adverse cardiovascular events (MACE) at 1 and 6 months after AMI. Methods: This prospective study included 150 consecutive patients with STEMI and NSTEMI enrolled between December 2024 and August 2025. MACE was defined as a composite of overall cardiac death, recurrent myocardial infarction, cerebrovascular insult, heart failure with reduced ejection fraction occurrence, and new revascularization, either PCI or CABG. Results: Patients with elevated Lp(a) had significantly greater coronary disease burden, reflected by higher mean SYNTAX scores (17.3 ± 7.0 vs. 13.8 ± 7.0; *p* = 0.011) and a greater proportion of intermediate- and high-risk SYNTAX classifications (*p* = 0.016). Although the number of diseased vessels did not differ significantly, three-vessel disease was more frequent in the elevated Lp(a) group. At 1-month follow-up, overall MACE incidence was numerically higher but not statistically significant between groups. At 6 months, heart failure with reduced ejection fraction was significantly increased in patients with elevated Lp(a) (27.7% vs. 12.2%; *p* = 0.027). Binary logistic regression demonstrated that elevated Lp(a) independently predicted 6-month MACE (OR 2.768, *p* = 0.011, 95% CI 1.262–6.072), but not 1-month outcomes. Conclusions: Elevated Lp(a) is associated with increased coronary artery disease severity and higher mid-term MACE risk after AMI.

## 1. Introduction

Cardiovascular disease (CVD) remains the leading cause of death worldwide and in Europe, and its burden is particularly pronounced in Bosnia and Herzegovina and the wider Southeast European region, where approximately half of all deaths are attributable to CVD, with coronary artery disease (CAD) accounting for nearly half of these cases [1]. Traditional cardiovascular risk factors are highly prevalent in Bosnia, with hypertension affecting approximately 41% of adults, smoking 35%, and obesity 33%, reflecting a substantial atherosclerotic burden; consequently, identifying residual risk factors beyond LDL cholesterol is essential in this setting to improve prognostic stratification following acute myocardial infarction (MI) [1,2]. Lipoprotein(a) [Lp(a)] is a cholesterol-rich, LDL-like particle bound to apolipoprotein(a), conferring pronounced pro-atherogenic, proinflammatory, and prothrombotic properties, and because its plasma concentration is largely genetically determined and remains relatively stable throughout adulthood, robust epidemiologic and genetic evidence over the past two decades has established Lp(a) as an independent causal risk factor for atherosclerotic cardiovascular disease (ASCVD) and calcific aortic stenosis [3,4]. In clinical practice, an Lp(a) level of ≥50 mg/dL (≈105 nmol/L), affecting approximately 20–25% of individuals, is widely considered a high-risk threshold, while levels above 30 mg/dL are already associated with increased cardiovascular risk, and large-scale cohort and Mendelian randomization studies consistently demonstrate a dose-dependent, linear relationship between higher Lp(a) concentrations and increased risk of ASCVD, coronary events, and death, independent of LDL cholesterol [5,6].

Numerous clinical studies have linked elevated Lp(a) to more severe CAD and worse outcomes. For instance, in a contemporary angiographic cohort, higher Lp(a) levels (and associated oxidized phospholipids) were significantly associated with multi-vessel CAD at baseline, and independently predicted follow-up major adverse cardiovascular events (MACE) [7]. Meta-analyses confirm this trend: one systematic review (*n* ≈ 560,000) found that high Lp(a) was associated with a ~26–33% higher risk of MACE in both primary and secondary-prevention populations, regardless of baseline hsCRP levels [8]. In survivors of acute MI, a recent pooled analysis (23 cohorts, ~30,000 patients) showed that individuals in the highest Lp(a) strata had modestly but significantly higher rates of subsequent MACE (adjusted HR ≈ 1.05–1.14) than those with low Lp(a) [9]. Similarly, in the PCSK9 inhibitor trials (FOURIER and ODYSSEY), baseline Lp(a) correlated with residual risk and with the magnitude of treatment benefit: patients with higher Lp(a) derived greater absolute reduction in events when Lp(a) was lowered by therapy [10]. Taken together, these data implicate Lp(a) as a contributor to both extensive coronary atherosclerosis and recurrent events, even after optimal LDL-C lowering.

Based on this evidence, recent high-impact guidelines now emphasize Lp(a) in risk assessment. The 2018 ACC/AHA cholesterol guideline considers Lp(a) ≥ 50 mg/dL a “risk-enhancing” factor that may reclassify intermediate-risk patients, particularly those with a family history of premature ASCVD [11,12]. The 2025 focused update of the ESC/EAS dyslipidemia guidelines goes further: it issues a Class IIa recommendation for at least one lifetime measurement of Lp(a) in all adults, and explicitly recognizes Lp(a) > 50 mg/dL as a risk modifier that should prompt more intensive risk management. These guidelines reflect growing consensus that Lp(a) screening is useful for identifying individuals who may benefit from aggressive therapy (e.g., PCSK9 inhibitors) and for informing novel Lp(a)-lowering trials [11,12].

Despite international interest, data from Southeast Europe are sparse. In Bosnia and Herzegovina, routine Lp(a) testing is uncommon, and no large studies have examined its impact on CAD severity or early post-MI outcomes. Yet the very high CVD mortality here argues that novel risk markers warrant study. Therefore, the objective of this study is to assess whether elevated Lp(a) levels are associated with greater angiographic CAD severity and with a higher incidence of MACE at 1 and 6 months following acute MI. By analyzing our regional cohort, we aim to clarify the prognostic value of Lp(a) in the critical early phase after MI and to inform regional risk stratification strategies.

## 2. Materials and Methods

### 2.1. Study Design and Protocol

A prospective cohort study was conducted on 150 non-diabetic consecutive patients aged ≥18 years, of both sexes, admitted with acute coronary syndrome who underwent invasive coronary angiography followed by percutaneous coronary intervention (PCI) with implantation of drug-eluting stents (DES). The study was carried out at the Clinic for Diseases of the Heart, Vessels and Rheumatism, Coronary unit, Clinical Center of the University of Sarajevo between December 2024 and August 2025. Patients meeting the inclusion criteria were consecutively enrolled during hospitalization for the index event (all-comers design). The sample size was not based on a formal a priori calculation but was determined by consecutive inclusion of all eligible non-diabetic patients with acute myocardial infarction admitted during the predefined study period. The study protocol was approved by the Ethics Committee of the Clinical Center of the University of Sarajevo, and all procedures were conducted in accordance with the Declaration of Helsinki.

### 2.2. Data Collection and Patient Groups

Patients were stratified into two groups according to lipoprotein(a) [Lp(a)] levels: <30 mg/dL and ≥30 mg/dL. The threshold of 30 mg/dL for Lp(a) was selected based on prior studies demonstrating that even moderately elevated Lp(a) levels are associated with increased cardiovascular risk. While a cut-off of 50 mg/dL is commonly used to define high-risk individuals, lower thresholds have also been shown to carry prognostic significance, particularly in patients with established cardiovascular disease. Baseline demographic data, cardiovascular risk factors, clinical presentation, laboratory parameters, angiographic findings, and in-hospital data were collected at admission. A venous blood sample (1–5 mL) was obtained by standard venepuncture at admission, together with routine laboratory parameters. Lp(a) levels were measured using a standardized turbidimetric assay on a Cobas analyser (Roche Diagnostics GmbH, Mannheim, Germany). Results were primarily expressed in nmol/L and subsequently converted to mg/dL. Lp(a) values were converted between mg/dL and nmol/L using a molar-mass-based approximation (1 mg/dL ≈ 2.5 nmol/L; 1 nmol/L ≈ 0.4 mg/dL). Coronary angiography was performed according to clinical indication, and the extent of coronary artery disease was assessed using the number of diseased vessels and the SYNTAX score. The SYNTAX score is an angiographic scoring system used to quantify the complexity and severity of coronary artery disease based on the number, location, and characteristics of coronary lesions, with higher scores indicating more complex and extensive disease. Left ventricular ejection fraction (LVEF) was indexed at the time of admission and reassessed at 1-month and 6-month follow-up using transthoracic echocardiography. HFrEF was defined as LVEF ≤ 40% in accordance with current guideline criteria and was evaluated at each time point to identify new-onset or worsening systolic dysfunction.

The primary outcome was the occurrence of major adverse cardiovascular events (MACE), defined as a composite of cardiovascular death (including sudden cardiac death), recurrent myocardial infarction, stroke, heart failure with reduced ejection fraction (NYHA class III–IV with echocardiographic confirmation), and repeat revascularization (PCI or coronary artery bypass grafting [CABG]). A new myocardial infarction was defined by typical clinical symptoms accompanied by a rise in troponin levels. Stroke was defined by neurological deficit with radiological confirmation. Cardiovascular death was defined as death resulting from a confirmed or probable cardiovascular cause based on clinical, laboratory, and radiological findings.

Patients were followed on an outpatient basis for six months, with two scheduled follow-up visits: approximately one month after the index procedure and at six months. Outcomes were assessed at both 1-month and 6-month follow-up visits.

Exclusion criteria included: previously diagnosed or newly diagnosed diabetes mellitus during the index ACS; ACS without obstructive coronary stenosis; patients referred for primary surgical revascularization (CABG); and patients receiving inclisiran or PCSK-9 inhibitors, as these therapies may significantly influence Lp(a) levels. Patients with diabetes mellitus were excluded due to inconsistent and often paradoxically reduced Lp(a) levels and the known association of diabetes with diffuse and advanced coronary artery disease, which could confound the analysis.

### 2.3. Statistical Analysis

Statistical analysis was performed using SPSS version 23. Normality of continuous variables was assessed prior to analysis, and depending on distribution, data are presented as mean ± SD with Student’s *t*-test or median (interquartile range) with Mann–Whitney U test, with appropriate use of non-parametric methods or transformation for skewed variables. Categorical variables were presented as frequencies and percentages and compared using the chi-square test. Binary logistic regression analysis was performed to identify independent predictors of MACE. Elevated Lp(a) was entered as the main independent variable. To account for potential confounding, the model was adjusted for clinically relevant covariates, including age, sex, LDL-C levels, clinical presentation (STEMI/NSTEMI), and renal function. Given the limited number of events, the number of covariates included in the model was restricted to reduce the risk of overfitting. A *p*-value < 0.05 was considered statistically significant.

## 3. Results

Patients were stratified into low Lp(a) (<30 mg/dL, N = 114) and elevated Lp(a) (>30 mg/dL, N = 36) groups. The proportion of males was higher in the low Lp(a) group compared with the elevated Lp(a) group (86.8% vs. 75.0%), without reaching statistical significance (χ^2^ = 2.85, *p* = 0.091). Mean age was similar between groups (59.7 ± 9.4 vs. 61.0 ± 12.1 years; *p* = 0.499). No significant differences were observed in cardiovascular risk factors. Hypertension was present in 74.5% vs. 66.7% (*p* = 0.354) and hyperlipidemia in 63.1% vs. 69.4% (*p* = 0.392) of patients in the low and elevated Lp(a) groups, respectively. Smoking was more frequent in the low Lp(a) group (71.9% vs. 58.3%), although not statistically significant (*p* = 0.125). A family history of cardiovascular disease was reported in 50.8% and 58.3% of patients, respectively (*p* = 0.435). All other data regarding baseline demographic and clinical characteristics are presented in Table 1.

Patients with elevated Lp(a) levels (>30 mg/dL, n = 36) more frequently presented with STEMI compared to those with low Lp(a) (<30 mg/dL, n = 114) (97.2% vs. 85.0%), reaching borderline statistical significance (*p* = 0.051).

There were no significant differences in hemodynamic parameters at admission. Systolic blood pressure was 145.4 ± 26.0 mmHg in the low Lp(a) group and 136.0 ± 21.5 mmHg in the elevated Lp(a) group (*p* = 0.213), while diastolic pressure showed a non-significant trend toward lower values in the elevated Lp(a) group (88.6 ± 16.3 vs. 84.5 ± 11.1 mmHg; *p* = 0.064). Heart rate was comparable between groups (77.6 ± 17.6 vs. 81.2 ± 21.5 bpm; *p* = 0.118).

Most hematological and biochemical parameters did not differ significantly between groups, including inflammatory markers, renal function, liver enzymes, cardiac biomarkers, electrolytes, and glycemic indices.

Significant differences were observed in lipid parameters. Patients with elevated Lp(a) had higher total cholesterol (7.4 ± 3.2 vs. 3.9 ± 1.9 mmol/L; *p* = 0.006), LDL cholesterol (4.7 ± 1.3 vs. 2.6 ± 1.9 mmol/L; *p* = 0.043), and non-HDL cholesterol (6.6 ± 3.3 vs. 2.8 ± 1.8 mmol/L; *p* = 0.012). HDL cholesterol and triglyceride levels were similar between groups. All other clinical, hemodynamic, and laboratory parameters are presented in Table 2.

Although the distribution of single-, two-, and three-vessel disease did not differ significantly between groups (*p* = 0.375), patients with elevated Lp(a) had a higher proportion of three-vessel disease. The mean SYNTAX score was significantly higher in the elevated Lp(a) group (17.3 ± 7.0 vs. 13.8 ± 7.0; *p* = 0.011). Similarly, SYNTAX risk stratification differed significantly between groups, with a lower proportion of patients classified as low risk and a higher proportion classified as intermediate and high risk in the elevated Lp(a) group (*p* = 0.016).

Regarding angiographic findings, the prevalence of left main, LAD, Cx, and RCA stenosis did not differ significantly between groups. Previous stent implantation was observed in 5.2% of patients in the low Lp(a) group and 8.3% in the elevated Lp(a) group (*p* = 0.472). All other angiographic findings and measures of coronary artery disease burden are presented in Table 3.

At 1-month follow-up, the incidence of individual MACE components did not differ significantly between the low and elevated Lp(a) groups. Although heart failure with reduced ejection fraction was more frequent in patients with elevated Lp(a) levels (25.0% vs. 15.7%), the difference was not statistically significant. Other endpoints, including overall cardiac death, recurrent myocardial infarction, cerebrovascular insults, repeated revascularization either with PCI or CABG, showed no significant intergroup differences.

At 6-month follow-up, heart failure with reduced ejection fraction was significantly higher in the elevated Lp(a) group compared with the low Lp(a) group (27.7% vs. 12.2%; *p* = 0.027). No statistically significant differences were observed for other MACE components, including overall cardiac death, recurrent myocardial infarction, cerebrovascular insults, PCI, or CABG. Binary logistic regression showed that the group of patients with elevated Lp(a) had no statistically higher rates of MACE at 1-month follow-up (*p* = 0.261), but significantly higher rates of MACE at 6-month follow-up (OR = 2.768, *p* = 0.011, 95% CI 1.262–6.072).

All other clinical outcomes and individual MACE components at 1- and 6-month follow-up are presented in Table 4.

## 4. Discussion

In this prospective cohort, we found that elevated Lp(a) was associated with more extensive CAD and with a significantly higher risk of composite MACE at 6 months. Notably, Lp(a) elevation did not predict very early (1-month) events in our cohort. These findings extend the observation that Lp(a) is a marker of residual risk in CAD—even in the acute setting—and suggest that high Lp(a) identifies patients with a greater atherosclerotic burden and poorer medium-term outcomes. This is broadly consistent with large-scale data showing continuous, independent associations between Lp(a) and CHD risk [13]. The continuous nature of risk was emphasized by Erqou et al. in a meta-analysis of 24 cohorts, which reported a risk ratio of ~1.13 (95% CI 1.09–1.18) for CHD per 1-SD higher Lp(a) after adjusting for other risk factors [13]. Our OR of ~2.77 for 6-month MACE in AMI patients with “high” Lp(a) (versus <30 mg/dL) reflects a much higher short-term risk increment, likely because our population was enriched for prevalent ASCVD and we used a dichotomous cut-off; nonetheless, the direction of risk (higher Lp(a) → higher events) is concordant with that meta-analysis. Moreover, recent meta-analyses focused specifically on ACS populations have similarly reported that high Lp(a) independently predicts recurrent events. For example, Jia et al. pooled >18,000 ACS patients and found that elevated Lp(a) conferred a 26% higher adjusted hazard of MACE (HR ≈ 1.26, 95% CI 1.17–1.35) [14]. This mirrors our findings and supports Lp(a) as a prognostic marker in the post-AMI setting.

Our results are consistent with the emerging literature linking elevated Lp(a) to CAD severity and worse outcomes after myocardial infarction. An updated meta-analysis by Liu et al. (2025) reported that higher Lp(a) was modestly but significantly associated with long-term MACE after AMI; notably, associations were stronger for events beyond the first year and among women and those with diabetes or hypertension [9]. We similarly saw that Lp(a) predicted events at 6 months but not at 1-month, suggesting that very early post-AMI events (likely driven by acute factors and infarct size) may be less influenced by Lp(a) than later events (which may reflect progressive atherothrombosis). In the context of these prior studies, our data add prospective evidence from an acute-care setting and a lower Lp(a) threshold (30 mg/dL) than some others.

Major trials of PCSK9 inhibitors in secondary prevention also provide indirect support for our findings [9,10]. In ODYSSEY Outcomes (recent-ACS patients on high-intensity statins) and FOURIER (stable ASCVD), modest reductions in Lp(a) due to evolocumab or alirocumab treatment were associated with significant relative reductions in MACE risk [15]. This suggests that Lp(a) contributes to residual risk beyond LDL-C, and that lowering it (even as an incidental effect of therapy) can improve outcomes. In ODYSSEY specifically, Bittner et al. showed that each 1 mg/dL drop in Lp(a) with alirocumab was independently associated with a 0.6% relative reduction in MACE risk (HR ≈ 0.994 per 1 mg/dL). These randomized-trial findings reinforce the biological relevance of Lp(a) that we observe in our cohort.

Current practice guidelines [11] also recognize Lp(a) as a cardiovascular risk factor. The 2018 ACC/AHA cholesterol guidelines include an Lp(a) ≥ 50 mg/dL (≈125 nmol/L) as a risk-enhancing factor favoring statin therapy. Likewise, the 2019 ESC/EAS dyslipidemia guidelines recommend measuring Lp(a) at least once in all adults, given its role as a genetic risk factor for ASCVD. These recommendations align with our implication that Lp(a) level contains prognostic information in patients with known CAD. In practice, however, Lp(a)-specific therapies are still investigational, so guidelines emphasize optimization of other risk factors (e.g., intensive LDL-lowering) for high-Lp(a) individuals.

Elevated Lp(a) may influence atherothrombotic risk through several pathophysiological pathways. Structurally, Lp(a) resembles an LDL particle bound to apolipoprotein(a); it carries a high load of cholesterol and oxidized phospholipids. These oxidized phospholipids are proinflammatory: Lp(a) particles can infiltrate the arterial intima, promote macrophage activation and apoptosis, and induce endothelial dysfunction, thus accelerating plaque growth and instability. The apo(a) moiety itself is homologous to plasminogen and interferes with fibrinolysis, contributing to a prothrombotic milieu. In sum, Lp(a) has been described as simultaneously pro-atherogenic, proinflammatory, and prothrombotic, which may explain why high Lp(a) predicts more extensive CAD and recurrent events.

This study has several limitations. First, it was a single-center observational cohort, which may limit generalizability; local patient demographics or practice patterns could bias the results. Second, the sample size and number of events were relatively modest, raising the possibility of type I or II error. Third, our follow-up was only 6 months; longer-term outcomes (beyond half a year) may reveal additional effects of Lp(a) not captured here. Fourth, we only measured Lp(a) once (at baseline); Lp(a) can behave as a mild acute-phase reactant, although studies show levels are relatively stable after the acute phase of MI.

Our findings suggest that measuring Lp(a) in patients after AMI could help identify those at higher risk of recurrent events. In line with current consensus, Lp(a) should be measured at least once in the lifetime of high-risk individuals. For post-AMI patients found to have elevated Lp(a), the intensity of secondary prevention should be maximized. This includes aggressive LDL-C lowering (high-intensity statins with or without ezetimibe or PCSK9 inhibitors) and strict control of other modifiable risks (blood pressure, glycemia, smoking cessation). Indeed, PCSK9 inhibitors may be particularly appealing in high-Lp(a) patients, since trials have shown they modestly lower Lp(a) (by ~20–30%) while reducing events. Even in the absence of Lp(a)-specific therapies, recognition of Lp(a) elevation justifies a more vigilant approach. In the near future, targeted Lp(a)-lowering agents (e.g., antisense oligonucleotides or siRNA against the LPA gene) are on the horizon, and high-risk patients identified by our findings could be candidates for such therapies once approved.

## 5. Conclusions

In conclusion, elevated Lp(a) in AMI patients marks a subgroup with more diffuse CAD and a higher likelihood of 6-month MACE. These results align with international evidence and underscore the importance of early risk stratification. Assessing Lp(a) adds prognostic information that can guide the intensity of post-AMI care: patients with high Lp(a) may benefit from especially aggressive lipid-lowering and risk-factor management to improve their clinical trajectory. Further large, multi-center studies with longer follow-up are warranted to confirm these findings and to refine how best to incorporate Lp(a) into secondary-prevention algorithms.

## Figures and Tables

**Table 1:** Baseline demographic and clinical characteristics according to lipoprotein(a) levels.

Variable		Low Lp(a) Group (<30 mg/dL) N = 114	Elevated Lp(a) Group (>30 mg/dL) N = 36	*p*-Value
Sex (N, %)	Male	99 (86.8)	27 (75.0)	0.091
	Female	15 (13.2)	9 (25.0)	
Age (mean ± SD)		59.7 ± 9.4	61.0 ± 12.1	0.499
Comorbidities (N, %)	Hypertension	85 (74.5)	24 (66.67)	0.354
	Hyperlipidemia	72 (63.1)	25 (69.4)	0.392
	Smoking	82 (71.9)	21 (58.3)	0.125
	Family history of cardiovascular disease	58 (50.8)	21 (58.3)	0.435

**Table 2:** Clinical presentation and laboratory findings according to lipoprotein(a) levels.

Variable		Low Lp(a) Group (<30 mg/dL) N = 114	Elevated Lp(a) Group (>30 mg/dL) N = 36	*p*-Value
Diagnosis (N, %)	STEMI	97 (85.0)	35 (97.2)	0.051
	NSTEMI	17 (15.0)	1 (2.8)	
Blood pressure (mean ± SD)	Systolic pressure	145.4 ± 26.0	136.0 ± 21.5	0.213
	Diastolic pressure	88.6 ± 16.3	84.5 ± 11.1	0.064
Heart rate (mean ± SD)		77.6 ± 17.6	81.2 ± 21.5	0.118
Laboratory parameters (mean ± SD)	WBC	11.8 ± 1.7	10.8 ± 2.9	0.589
	Hgb	145.7 ± 8.0	145.0 ± 27.2	0.124
	Hct	44.7 ± 3.2	44.0 ± 8.1	0.173
	RDW	13.5 ± 0.4	14.7 ± 1.5	0.599
	PLT	233.7 ± 17.1	272.3 ± 72.9	0.862
	CRP	4.1 ± 1.3	5.7 ± 0.7	0.469
	Urea	6.0 ± 2.4	7.3 ± 1.8	0.364
	Creatinine	74.0 ± 24.5	107.3 ± 27.4	0.895
	eGFR	87.0 ± 19.7	67.0 ± 13.4	0.594
	CK	558.7 ± 587.7	152.0 ± 108.0	0.913
	CKMB	93.7 ± 9.7	37.3 ± 30.0	0.687
	Hs-Troponin	719.2 ± 1086.5	19.6 ± 10.5	0.474
	Total cholesterol	3.9 ± 1.9	7.4 ± 3.2	0.006
	LDL cholesterol	2.6 ± 1.9	4.7 ± 1.3	0.043
	HDL cholesterol	1.1 ± 0.07	0.8 ± 0.08	0.693
	Non-HDL cholesterol	2.8 ± 1.8	6.6 ± 3.3	0.012
	Triglycerides	1.2 ± 0.6	4.9 ± 4.7	0.513
	Hb1AC%	6.0 ± 0.3	5.8 ± 0.05	0.087

**Table 3:** Extent of coronary artery disease and angiographic findings according to lipoprotein(a) levels.

Variable		Low Lp(a) Group (<30 mg/dL) N = 114	Elevated Lp(a) Group (>30 mg/dL) N = 36	*p*-Value
Number of diseased vessels (N, %)	Single-vessel disease	46 (40.3)	11 (30.5)	0.375
	Two-vessel disease	40 (35.0)	13 (36.1)	
	Three-vessel disease	28 (24.7)	12 (33.4)	
Syntax score (mean ± SD)		13.8 ± 7.0	17.3 ± 7.0	0.011
Syntax grade (N, %)	Low risk	90 (78.9)	20 (55.5)	0.016
	Intermediate risk	19 (16.6)	13 (36.1)	
	High risk	5 (4.5)	3 (8.4)	
Coronary angiography findings (N, %)	Left main stenosis	2 (1.7)	0 (0.0)	0.430
	LAD stenosis	80 (70.1)	28 (77.7)	0.255
	Cx stenosis	55 (48.2)	20 (55.5)	0.357
	RCA stenosis	75 (65.7)	23 (63.8)	0.993
	Previous stents	6 (5.2)	3 (8.3)	0.472

**Table 4:** Major adverse cardiovascular events at 1-month and 6-month follow-up according to lipoprotein(a) levels.

Variable		Low Lp(a) Group (<30 mg/dL) N = 114	Elevated Lp(a) Group (>30 mg/dL) N = 36	*p*-Value
MACE at 1-month follow-up (N, %)	Overall cardiac death	3 (2.6)	2 (5.4)	0.394
	Myocardial infarction	3 (2.6)	1 (2.7)	0.962
	Cerebrovascular insult	4 (3.5)	0 (0.0)	0.254
	Heart failure occurrence	18 (15.7)	9 (25.0)	0.209
	PCI	12 (10.5)	3 (8.3)	0.702
	CABG	0 (0.0)	1 (2.7)	0.074
MACE at 6-month follow-up (N, %)	Overall cardiac death	1 (0.8)	1 (2.7)	0.386
	Myocardial infarction	1 (0.8)	0 (0.0)	0.572
	Cerebrovascular insult	1 (0.8)	2 (5.4)	0.080
	Heart failure occurrence	14 (12.2)	10 (27.7)	0.027
	PCI	9 (7.8)	5 (13.8)	0.281
	CABG	6 (5.2)	2 (5.4)	0.945

## Data Availability

The data presented in this study are available on request from the corresponding author. The data are not publicly available due to privacy and anonymity restrictions.
